# The Microenvironment in an Experimental Model of Acute Pancreatitis Can Modify the Formation of the Protein Corona of sEVs, with Implications on Their Biological Function

**DOI:** 10.3390/ijms252312969

**Published:** 2024-12-02

**Authors:** Olga Armengol-Badia, Jaxaira Maggi, Carme Casal, Roser Cortés, Joaquín Abián, Montserrat Carrascal, Daniel Closa

**Affiliations:** 1 Department of Experimental Pathology, Institut d’Investigacions Biomèdiques de Barcelona, Consejo Superior de Investigaciones Científicas (IIBB-CSIC), Institut d’Investigacions Biomèdiques August Pi i Sunyer (IDIBAPS), 08036 Barcelona, Spain; olga.armengol@iibb.csic.es (O.A.-B.); roser.cortes@iibb.csic.es (R.C.); 2 Doctorate in Biotechnology, Facultat de Farmàcia i Ciències de l’Alimentació, Universitat de Barcelona, 08028 Barcelona, Spain; 3 Biological and Environmental Proteomics, Institut d’Investigacions Biomèdiques de Barcelona, Consejo Superior de Investigaciones Científicas (IIBB-CSIC), Institut d’Investigacions Biomèdiques August Pi i Sunyer (IDIBAPS), 08036 Barcelona, Spain; jaxaira.maggi@iibb.csic.es (J.M.); joaquim.abian.csic@uab.cat (J.A.); montserrat.carrascal.csic@uab.cat (M.C.); 4 Microscopy Unit, Institut d’Investigacions Biomèdiques de Barcelona, Consejo Superior de Investigaciones Científicas (IIBB-CSIC), 08036 Barcelona, Spain; carme.casal@iibb.csic.es

**Keywords:** extracellular vesicles, protein corona, inflammation, acute pancreatitis, exosomes

## Abstract

A considerable number of the physiological functions of extracellular vesicles are conditioned by the protein corona attached to their surface. The composition of this corona is initially defined during their intracellular synthesis, but it can be subsequently modified by interactions with the microenvironment. Here, we evaluated how the corona of small extracellular vesicles exposed to the inflammatory environment generated in acute pancreatitis is modified and what functional changes occur as a result of these modifications. Small extracellular vesicles obtained from a pancreatic cell line were incubated with the ascitic fluid generated in experimental acute pancreatitis in rats. Using proteomic techniques, we detected the appearance of new proteins and an increase the uptake of extracellular vesicles by certain cell types and the response induced in inflammatory cells. The inhibition of different pattern recognition receptors reversed this activation, indicating that some of these effects could be due to binding of damage-associated molecular patterns to the corona. All of this indicates that in pathologies such as acute pancreatitis, characterized by an inflammatory response and intense tissue damage, the microenvironment substantially influences the corona of extracellular vesicles, thus altering their behavior and enhancing their inflammatory activity.

## 1. Introduction

Small extracellular vesicles (sEVs), often called exosomes, represent a subtype of extracellular vesicles typically measuring around 150 nm in size and are smaller than other extracellular vesicles, such as apoptotic bodies or microvesicles [1]. Since their discovery, sEVs have emerged as integral players in intercellular communication, transporting functional proteins, mRNA, and microRNA. Upon fusion with target cells, sEVs release their cargo into the cytoplasm, facilitating essential cell-to-cell communication pathways. In pathological contexts, sEVs can contribute to the onset or perpetuation of diseases, and their involvement in a spectrum of pathologies underscores their potential as biomarkers and therapeutic agents [2,3,4].

Although sEVs are commonly viewed as vesicles that transport mediators between cells, it should be noted that their function extends far beyond being simple containers. The surface of sEVs is covered in a complex and dynamic protein corona that modulates some of their biological functions [5,6]. Given their small size, the surface-to-volume ratio is extremely high, so more proteins can be attached to the surface of these nanovesicles than inside the lumen [7]. Much of this corona is formed during secretion, and its composition depends on the source cell but, once released, adsorptive interactions between the vesicle surface and soluble constituents of the surrounding biological fluid can modify its composition [8]. Furthermore, as sEVs travel through different biological fluids, they rapidly accumulate additional proteins on their surfaces. This can be relevant in microenvironments associated with the inflammatory response or tissue damage. In these areas, sEVs encounter a myriad of biomolecules that can adhere to their surface and modify the composition and characteristics of their protein corona, thus altering their biological function [9].

In this study, we evaluated the impact of the microenvironment on the formation of the protein corona of sEVs, using the inflammatory microenvironment generated during acute pancreatitis as a model. This disease, in its severe form, is characterized by rapid progression towards a process of systemic inflammation, in which sEVs have recently been seen to play a prominent role [10,11]. Although it is generally assumed that the effects of sEVs are due to the mediators they carry internally, it is also possible that their formation in an inflammatory environment influences their protein corona, which may also contribute to extending the inflammatory response to distant areas. In this case, the control of the changes experienced by the corona of sEVs from different microenvironments would become a relevant therapeutic target.

## 2. Results

### 2.1. Characterizing sEVs

**Figure 1:**
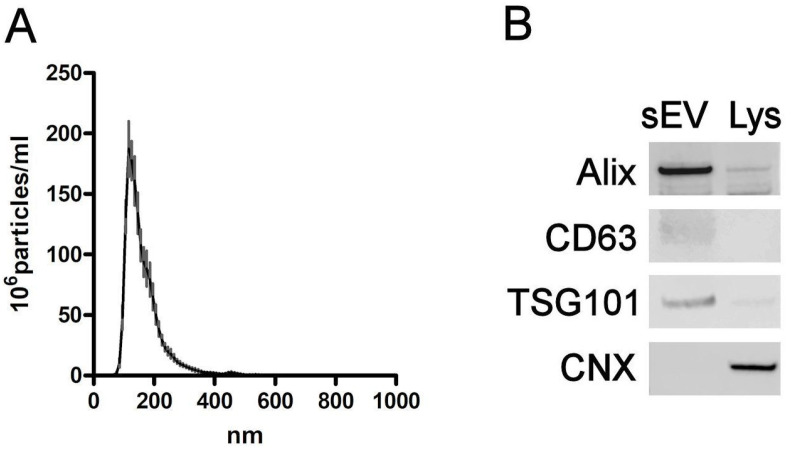

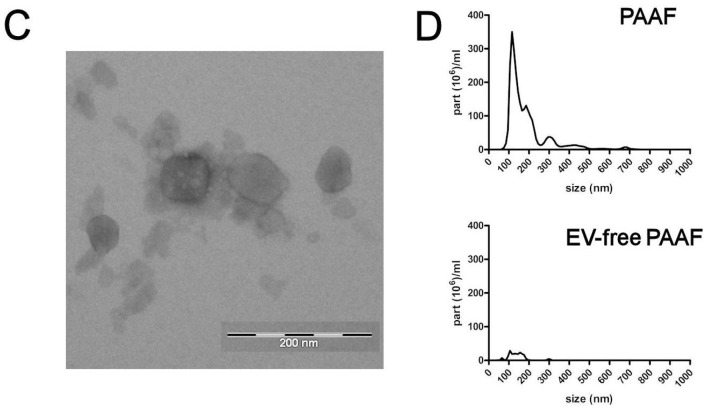
(**A**) Nanoparticle tracking analysis of sEVs obtained from BXPC3 cells. (**B**) sEVs and cell lysates (Lys) were analyzed via WB for TSG101, CD63 and Alix (EV biomarkers) and calnexin CNX (cellular biomarker). (**C**) TEM image of BXPC3-derived sEVs. (**D**) Nanoparticle tracking analysis of PAAF before (top) and after (bottom) removal of sEVs.

A nanoparticle tracking assay confirmed that the size of the particles obtained, around 150 nm, is in agreement with the expected size for sEVs (Figure 1A). Western blot confirmed the presence of the exosome markers Alix, CD63 and TSG101, as well as the absence of calnexin (Figure 1B). Images from transmission electron microscopy showed the typical morphology and size of sEVs (Figure 1C). Finally, a nanoparticle tracking assay confirmed the removal of endogenous extracellular vesicles from PAAF (Figure 1D).

### 2.2. Hydrodynamic Size and z-Potential

The incubation of sEVs with PAAF resulted in changes inn their z-potential, which became more electronegative (Table 1). On the other hand, there were no significant changes in the hydrodynamic size of sEVs. The small increase in both mode and mean after incubation with PAAF did not achieve statistical significance.

### 2.3. Cell Uptake

**Figure 2:**
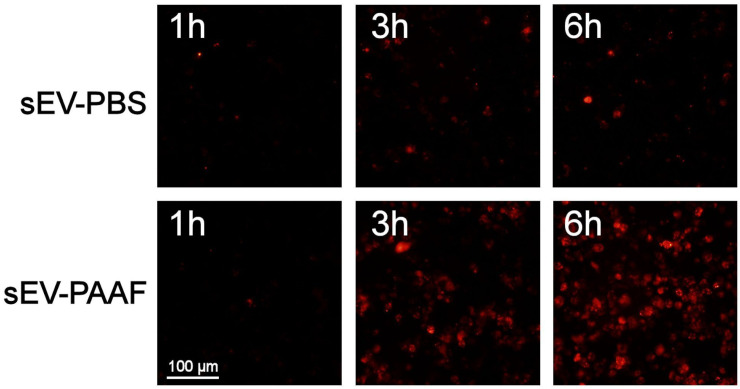
The kinetics of the uptake of sEVs by THP1 macrophages revealed that the rate at which they were taken up was greatly increased if they had been pretreated with PAAF. sEVs were stained red with PKH26.

In the first experiment, we measured how incubation with PAAF modified the uptake of sEVs by macrophages. The PAAF-treated sEVs incorporated faster than the control sEVs (incubated with PBS) (Figure 2). A difference could already be detected at one hour of incubation and was much more evident at six hours.

**Figure 3:**
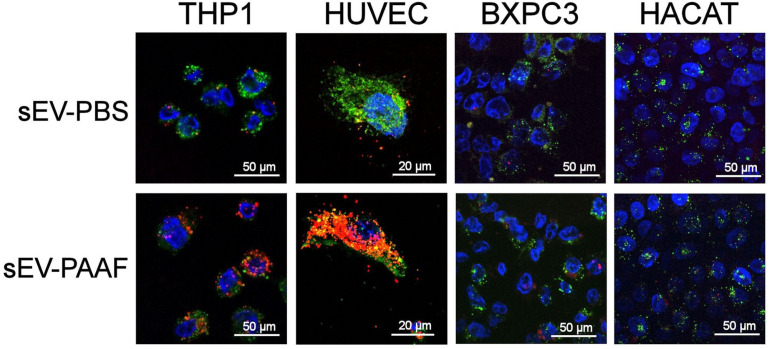
An increase in the uptake of sEVs after being treated (6 h) with PAAF was detected in macrophages (THP1) and in endothelial cells (HUVEC). In contrast, in the case of epithelial cells (BXPC3) and keratinocytes (HaCaT), no changes in uptake level were observed. sEVs were labeled with PKH26 Red Fluorescent Cell Linker Dye, cells were labeled with PKH67 Green Fluorescent Cell Linker Dye, and the cell nucleus was stained blue with Hoechst33342.

To see if this increased uptake was a general mechanism or was specific to macrophages, we analyzed the uptake in different cell lines after six hours of incubation. An increase induced by incubation with PAAF was detected in HUVEC endothelial cells and macrophages. In contrast, no change was observed in BXPC3 pancreatic epithelial cells or HaCaT keratinocytes (Figure 3).

### 2.4. Changes in Macrophage Phenotype and Gene Expression

**Figure 4:**
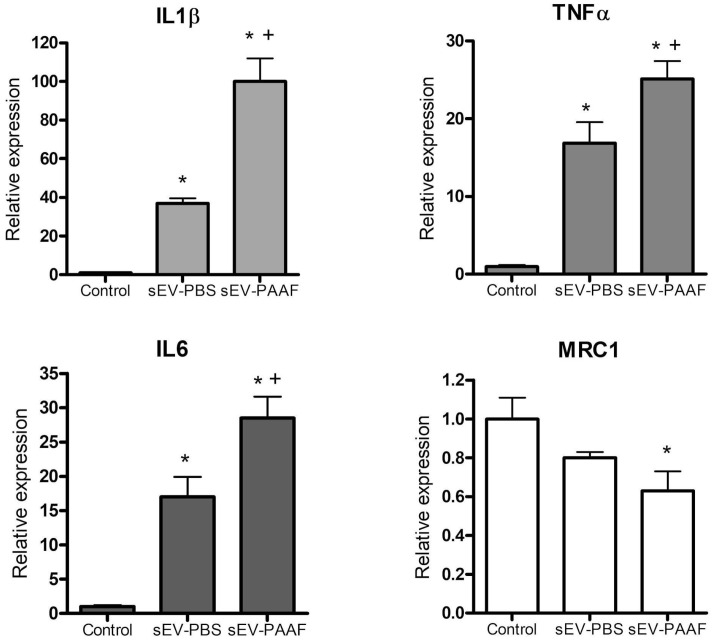
Changes in the expression of IL1β, TNFα, IL6 and MRC1 on THP1 macrophages incubated for 24 h with sEVs (10 µg/mL) pretreated with PBS (sEV-PBS) or PAAF (sEV-PAAF). * = *p* < 0.05 vs. control; + = *p* < 0.05 vs. sEV-PBS.

To assess whether the incubation of sEVs with PAAF modified the effect they had on the polarization of macrophages, the expression of genes linked to the M1 (IL1β and TNFα) and M2 (MRC1) responses was measured by RT-PCR after 24 h incubation with sEVs (Figure 4). Treatment with control sEVs (sEV-PBS) resulted in an increase in the expression of IL1β, IL6 and TNFα but did not modify MRC1 expression. When sEVs were treated with PAAF (sEV-PAAF), an additional increase in the expression of IL1β, IL6 and TNFα was detected, while MRC1 was inhibited.

### 2.5. Activation of Pattern Recognition Receptors (PRRs) by the Corona of sEVs

**Figure 5:**
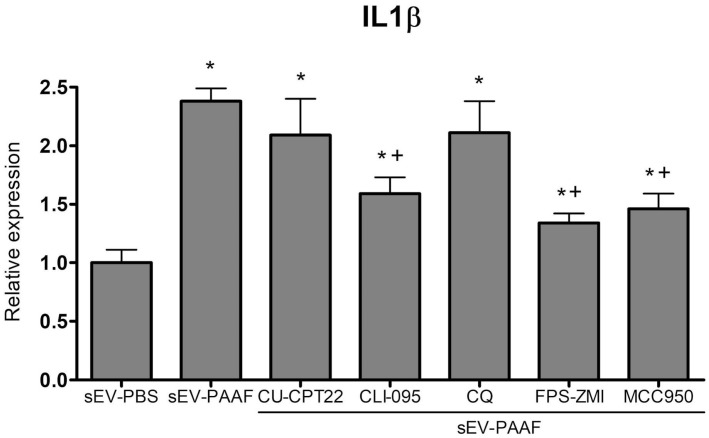
The increase in the expression of IL1β on THP1 macrophages induced by PAAF-pretreated sEVs was partially prevented by treating the cells with the TLR4 inhibitor CLI-095, the RAGE inhibitor FPS-ZMI or the inflammasome inhibitor MCC950. By contrast, no effect was observed with the inhibitors of TLR3,7,8,9 Chloroquine (CQ) and TLR 1,2 CU-CPT22. * = *p* < 0.05 vs. sEV-PBS; + = *p* < 0.05 vs. sEV-PAAF.

To determine the involvement of PRRs in the response to changes in the corona of sEVs, we evaluated the effect of different inhibitors on the induction of IL1β expression (Figure 5). Again, treating sEVs with PAAF induced an increase in IL1β expression. However, when macrophages were treated with inhibitors of TLR4 (CLI-095), RAGE (FPS-ZMI) or the NLRP3 inflammasome (MCC950), this induction was partially inhibited. In contrast, the inhibition of TLR 3,7,8,9 (CQ) or TLR 1,2 (CU-CPT22) had no effect.

### 2.6. Changes in Composition of Proteins in Corona of sEVs

To analyze the changes in the composition of the initial protein corona after PAAF incubation, we carried out a shotgun proteomics study of proteins exposed on the surface of PAAF-treated sEVs and PBS-treated ones as control. A total of 2051 proteins (1% FDR, >1 PSM, excluding keratins) were identified (Supplementary Table S2); only proteins with at least two unique peptides and those detected in at least two samples were considered for further analyses. We took advantage of the fact that PAAF was obtained from rat cells while sEVs came from human cells to identify the origin of the proteins. Although it is not always possible to differentiate between rat and human proteins due to the high homology of some of them, we were able to unequivocally identify 74 rat proteins, based on unique peptides. These proteins are considered to be bound to the sEV corona after incubation with PAAF (Table 2). Of these, there are 10 that represent 50% of the total abundance of proteins added to the corona. Predictably, these mainly include proteins related to plasma, such as complement C3, hemoglobin or fibrinogen, but the list also includes proteins secreted by the pancreas, such as lipase or pancreatic elastase, and others from different origins, probably coming from cell necrosis associated with pancreatitis.

**Figure 6:**
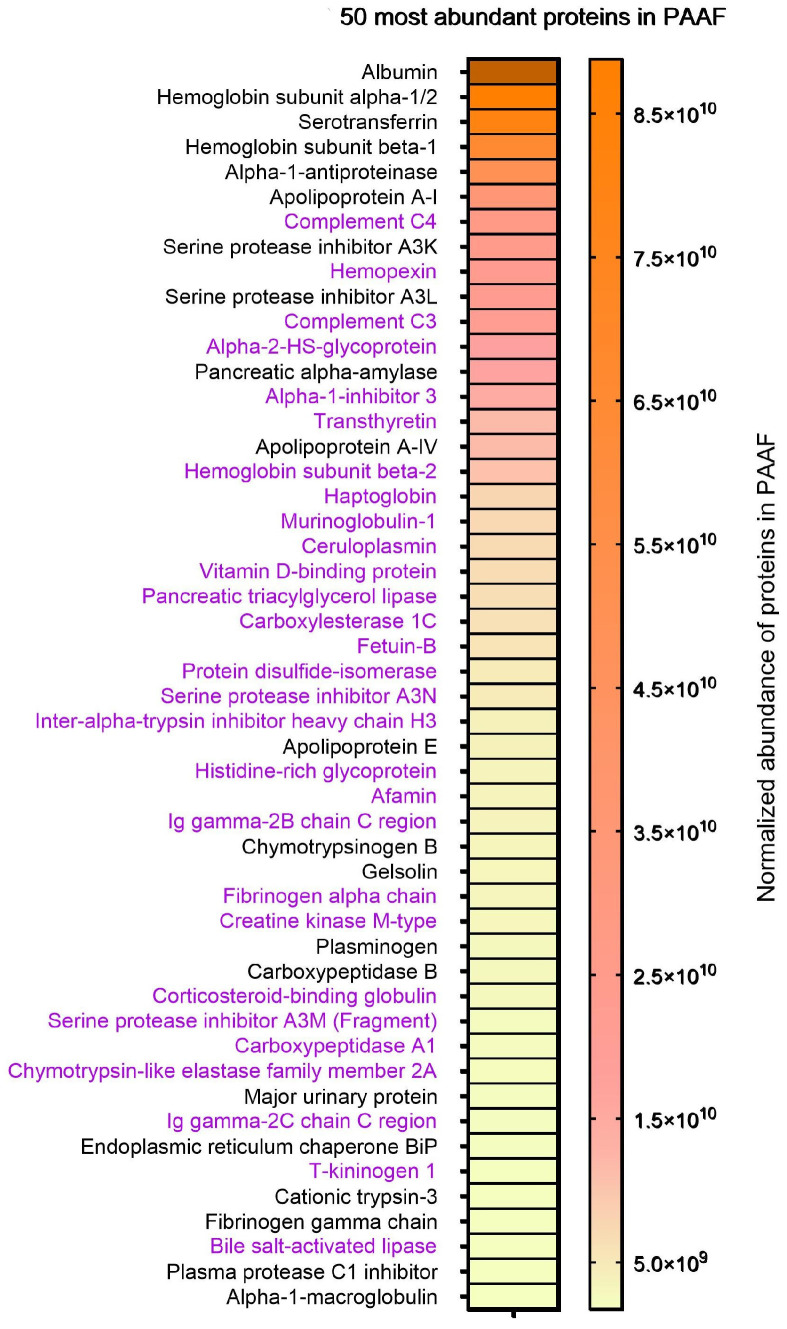
A heatmap of the 50 most abundant proteins in PAAF. The data are presented as the normalized abundance of proteins in PAAF. The range of values used in the graph is 1 × 10^9^–8.8 × 10^10^. The values outside the defined range are filled in dark orange. Proteins that were also found in PAAF-incubated sEVs are highlighted in purple.

A proteomic analysis of PAAF was also carried out (Supplementary Table S3). A heatmap of the 50 most abundant proteins in PAAF (Figure 6) revealed that many of those identified in the corona of the PAAF-incubated sEVs were among the most abundant (purple-highlighted proteins in Figure 6). However, there is no direct correlation between the relative abundance in PAAF and the coronal presence of PAAF-treated sEVs.

### 2.7. Protein–Protein Interaction Network

**Figure 7:**
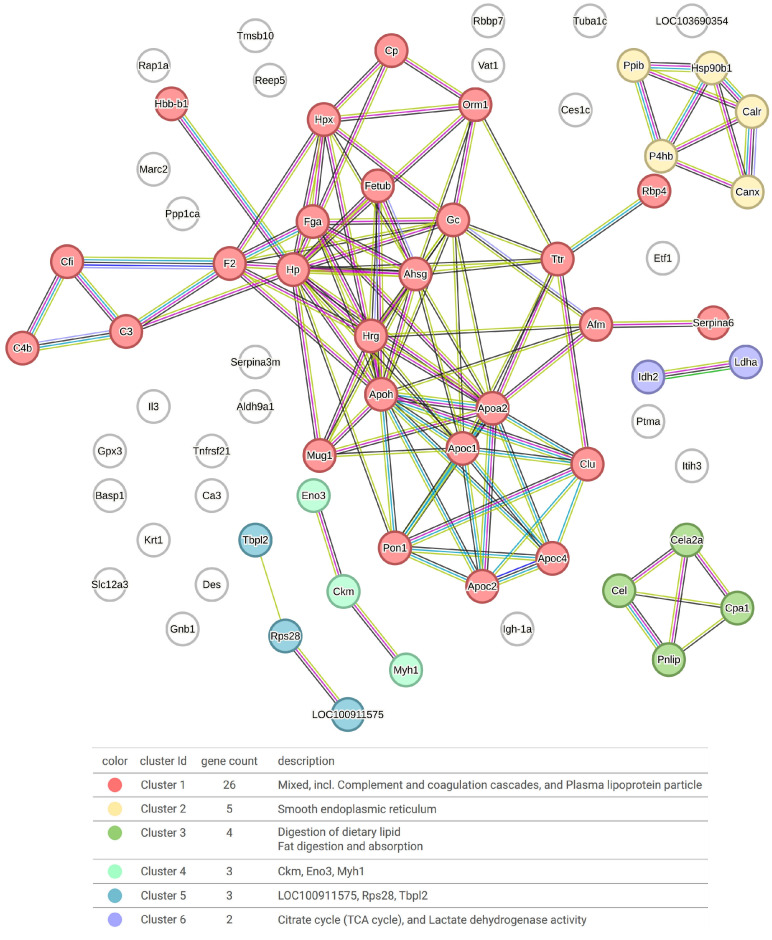
STRING interaction analysis of the proteins bound to the corona of PAAF-treated sEV samples. The line color indicates the type of interaction evidence.

Protein–protein interaction networks of upregulated proteins in the corona of PAAF-treated sEVs were constructed via the Cytoscape platform using the String application. The network mapping of proteins uniquely attributed to a rat origin revealed a high degree of connectivity in a cluster mainly related to the coagulation cascade, the complement system and lipoproteins (Figure 7). All of these factors are related to plasma, since a large part of PAAF is generated by the extravasation of plasma in places where vascular permeability has been affected by pancreatic damage. Minor clusters are related to digestive enzymes or the smooth endoplasmic reticulum.

### 2.8. Gene Ontology Analysis of Proteins Upregulated in PAAF-Treated sEVs

**Figure 8:**
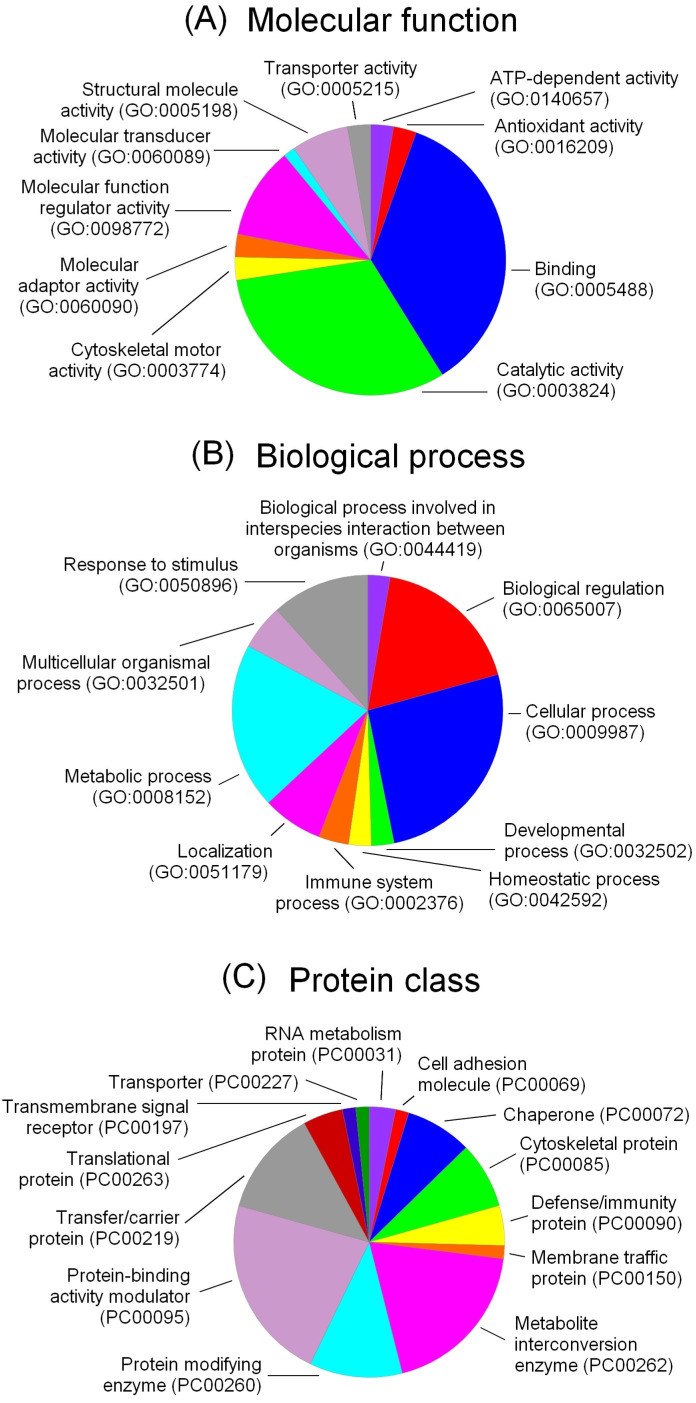
Frequency of proteins associated with different molecular functions (**A**), biological processes (**B**) and protein classes (**C**), based on the Gene Ontology database annotations for proteins on the surface of PAAF-treated sEV samples.

To elucidate the comprehensive functional enrichment features of PAAF-derived proteins upregulated in the corona of PAAF-treated sEVs, a GO enrichment analysis was conducted based on the annotations from the PANTHER database. The upregulated proteins were classified according to their molecular functions, biological roles and protein class. The molecular function analysis showed significant enrichment in binding, regulator, and catalytic activity (Figure 8A). For the biological process category, the main enrichment was observed for biological regulation, cellular processes and metabolic processes (Figure 8B). Finally, the analysis revealed that protein-binding, metabolite interconversion enzyme and transfer/carrier protein activity modulator proteins were the most representative classes among the group (Figure 8C).

## 3. Discussion

It is widely accepted that the surface corona of sEVs plays an important role in their function and that, in addition to the initial coat generated during the synthesis of the sEVs themselves, the microenvironment where they are released will also provide new proteins and elements that will end up configuring the final protein corona. In this work, we have shown that sEVs generated by pancreatic cells can experience important changes in their physiological effects when exposed to the inflammatory microenvironment generated during acute pancreatitis.

In the composition of the protein corona of nanomaterials, different layers are distinguished depending on their affinity, the intensity of the interactions and the speed of formation [12]. The hard corona involves the most intense interactions, and its formation occurs very quickly, in a matter of minutes. In contrast, the time periods required for the soft corona to form can be hours. The in vivo half-life of EVs has been reported to be less than 30 min [13], so the interactions and effects associated with the soft corona are likely not very significant in terms of physiological effects. For this reason, we focused on the changes that take place at the level of the hard corona and limited the incubation period of the sEVs with the PAAF to five minutes.

This short time of incubation with PAAF was enough to change the z-potential of sEVs, as might be expected from the binding of new proteins to the corona (Table 1). The reduction in the z-potential resulted in higher stability of nanoparticles, in line with other works that have described that plasma proteins, such as albumin, increase the stability of sEVs [14].

One of the most notable effects of corona modification is on cellular uptake. Macrophages take up sEVs more and more quickly after they have been exposed to PAAF (Figure 2). This increase in uptake is also clearly detected in HUVEC cells, whereas in BXPC3 or HaCaT cells, it is almost unappreciable (Figure 3), indicating that it is not a general phenomenon but must be associated with the presence of new proteins on the corona that act as additional ligands for cellular receptors [8]. This change is important since it indicates not only an increase in uptake but also a change in what the target cells will be before and after passing through a medium like PAAF.

The exposure of sEVs to the inflammatory microenvironment of acute pancreatitis also had relevant consequences regarding their biological effects. Macrophages treated with unexposed sEVs already resulted in an increase in the expression of different cytokines as a consequence of the inherent properties of sEVs (Figure 4). In our model, the sEVs were collected from cells maintained in the absence of FCS in the culture medium in order to ensure that they only carries the part of the protein corona generated during synthesis inside the cell and also to avoid the interference of the proteins already present in the serum [15]. This implies that the progenitor cells were subjected to a certain level of stress that could be reflected in the initial activity of the sEVs [16]. However, when incubated with PAAF (sEV-PAAF), the expression level of IL1β, TNFα and IL6 showed additional increases, while MRC1 expression levels were decreased. These data suggest that exposing sEVs to an inflammatory microenvironment causes them to induce macrophages to exhibit a more intense shift towards an inflammatory M1 phenotype.

In macrophages, most of these interactions are mediated by different PRRs [17,18]. In the case of PAAF, this is predictably to be linked to the presence of different damage-associated molecular pattern molecules (DAMPs), since, along with the inflammatory process, pancreatitis is associated with intense tissue damage. Inflammation is associated with a certain level of cell death and the generation of DAMPs, but in pancreatitis, this effect is more intense due to the presence of hydrolytic enzymes released by damaged pancreatic acinar cells. To determine whether PRRs were involved in the activation of macrophages in response to changes in the sEV corona, we treated them with inhibitors of different PRRs. The results (Figure 5) indicated that blocking TLR4 and RAGE prevented the increase in IL1β expression detected in response to PAAF treatment. While endosomal PRRs (TLR 3, 7, 8 and 9) might have been expected to be involved in the response, given their role in the intracellular trafficking of sEVs, inhibition with CU-CPT22 had no effect. However, the specific type of PRR inhibition observed in our experiment cannot be generalized, as the corona will be enriched with different proteins and biomolecules in response to different microenvironments, which may engage different TLRs. What our results confirm is that there are PRRs that recognize the markers added to the corona after the generation of sEVs, and this modifies the cell’s response to these vesicles.

The proteomic analysis of the corona of sEVs treated with PAAF revealed the appearance of new proteins that predictably adhered to the surface of the vesicle. We identified 74 proteins that appeared in the corona of sEVs incubated with PAAF. Although this represents a relatively small percentage compared to those already present in the corona, it should be noted that due to the high homology between rats and humans, there are proteins that could not be unequivocally assigned to rats and were therefore excluded from the list, so the number of PAAF proteins added to the corona must be even greater. As expected, many of the proteins added to the corona were plasma proteins. This became very evident when performing the interaction analysis, which revealed a large cluster of plasma-related proteins (Figure 7). However, there was also a significant number of proteins from other origins.

Another interesting fact is the comparison between the relative abundances of the proteins present in the PAAF and those in the corona of the sEVs (Figure 6 and Table 2). Obviously, the profile of new proteins attached to the corona does not simply reflect their abundance in the PAAF, and the interactions due to the physico-chemical characteristics of each protein are much more relevant. This means that although most upregulated proteins are found in some abundance in PAAF, there are also very abundant ones that do not end up binding to sEVs.

The analysis of the molecular functions and biological processes associated with the regulated proteins (Figure 8) allows us to understand the changes observed in the response of cells treated with sEVs after being exposed to PAAF. Most of these proteins are involved in regulating molecular and biological functions. A relevant subset is linked with catalytic activity, part of which appears to be due to the presence of some pancreatic enzymes released during pancreatitis, such as lipase or chymotrypsinogen. Notably, some of the novel proteins, such as HRG or calreticulin, have long been recognized to play a role in the damage-associated molecular pattern (DAMP) response. This suggests that circulating through an injured area of the body or a site with severe inflammatory damage can transform some initially unresponsive sEVs into DAMP transporters, helping to spread the inflammatory response to distant sites. This is consistent with the fact that part of the macrophage response to PAAF-treated sEVs is associated with the activation of some of the pattern recognition receptors (PRRs) (Figure 5).

It is important to note that, although our results indicate that the microenvironment significantly influences the final composition of the sEV protein corona, the specific changes observed are limited to our experimental model, which employs PAAF as a representation of an inflammatory microenvironment. It is evident that other microenvironments generated under different conditions are also likely to affect the sEV corona but would result in distinct profiles with potentially different impacts on their biological function.

## 4. Materials and Methods

### 4.1. Cells and Cell Culture

Human pancreatic BXPC3 and Keratinocyte HaCaT cell lines (ATCC, Manassas, VA, USA) were maintained in DMEM (Merck, Darmstadt, Germany) containing 10% fetal bovine serum (FBS; Thermo Fisher Scientific, Waltham, MA, USA), 100 U/mL penicillin and 100 µg/mL streptomycin. Human THP-1 cells (ATCC) were cultured in suspension in RPMI 1640 medium supplemented with 10% FCS, 2 mM L-glutamine, 100 U/mL penicillin, 100 µg/mL streptomycin and 2.5 μg/mL amphotericin. Cells were differentiated to macrophages through incubation with 100 nM phorbol 12-myristate 13-acetate (PMA) (Merck, Darmstadt, Germany) for 24 h. HUVECs (PromoCell, Heidelberg, Germany) were cultured in endothelial cell growth medium (ECGM; PromoCell, Heidelberg, Germany) containing 10% FCS (PromoCell, Heidelberg, Germany), EGM-2 SingleQuots (Lonza, Basel, Switzerland), hEGF, heparin, hydrocortisone and ascorbic acid.

### 4.2. Isolation of sEV

To isolate BXPC3-derived sEVs, cells were transferred into serum-free medium for 72 h in T-175 flasks. The medium was harvested and centrifuged sequentially at 3000× *g* 10 min and 10,000× *g* 30 min and filtered through 0.22 μm sterile filters (Millipore, Burlington, MA, USA). Subsequently, EVs were pelleted by ultracentrifugation at 100,000× *g* and 4 °C for 2 h, washed by resuspending them in 25 mL of PBS and ultracentrifuged again at 100,000× *g* for two more hours in order to remove possible remnants of soluble proteins; finally, they were resuspended in PBS. Total protein was quantified using a Pierce BCA Protein Assay Kit (ThermoFisher Scientific, Waltham, MA, USA).

### 4.3. Nanoparticle Tracking Analysis and z-Potential Measurement

The size distribution and the concentration of sEVs were measured using a NanoSight LM10 machine (NanoSight, Malvern, UK). The background was measured by testing filtered PBS, which revealed no signal. The zeta potential of the sEVs was measured using a Zetasizer Nano ZS (Malvern Instruments, Malvern, UK).

### 4.4. SDS-PAGE and Western Blot

sEV proteins were extracted in RIPA buffer supplemented with protease inhibitors. Proteins were separated on a 4–12% SDS-PAGE (Invitrogen™, NuPAGE™ 4 to 12%, Bis-Tris, 1.0–1.5 mm, Thermo Fisher, Waltham, MA, USA), transferred onto a PVDF membrane (Immuno-Blot, Bio Rad, Hercules, CA, USA) and blocked for 1 h in 5% nonfat milk in TBST, followed by overnight incubation at 4 °C with antibodies against TSG101 (14497-1-AP), ALIX (12422-1-AP), CD63 (25682-1-AP) and Calnexin (10427-2-AP) (ProteinTech, Rosemont, IL, USA). The blots were washed and incubated for 1 h at room temperature with DyLight 800-conjugated secondary antibodies (925-32211) (LI-COR Biosciences, Lincoln, NE, USA). Immunoreactive bands were visualized using an Odyssey Infrared Imaging System, Image Lab Touch Software version 2.3.0.07 (LI-COR Biosciences, Lincoln, NE, USA).

### 4.5. Electron Microscopy

Isolated extracellular vesicles were fixed in 4% paraformaldehyde, adsorbed in formvar-coated copper grids and negatively stained with 2% uranyl acetate. The grids were air dried and observed with a JEOL-1010 Transmission Electron Microscope (Jeol, Akishima, Japan). 

### 4.6. Animal Model of Acute Pancreatitis

Male Wistar rats (250 g b.w.) (Charles River, Lyon, France) were fed with standard laboratory pelleted formula A04 (Panlab, Barcelona, Spain) and tap water ad libitum. AP was induced through retrograde perfusion of 5% sodium taurocholate (Sigma, St. Louis, MO, USA). The control animals received an infusion of saline solution. Six hours after induction, pancreatitis-associated ascitic fluid (PAAF) was collected and processed immediately.

### 4.7. Production of EV-Depleted PAAF

PAAF was centrifuged at 3000× *g* 10 min and 10,000× *g* 30 min and diluted to 20% in PBS. Next, it was centrifuged at 100,000× *g* for 16 h. The supernatant was aliquoted, and the depletion of EV was confirmed by nanoparticle tracking analysis.

### 4.8. Incubation of sEVs with EV-Depleted PAAF

A total of 100 μL of BXPC3-derived sEVs were incubated with 100 μL of EV-depleted PAAF (sEV-PAAF) or PBS (sEV-PBS) for 5 min. Then, the mixture was washed by adding 20 mL of cold PBS, and the sEVs were pelleted via ultracentrifugation at 100,000× *g* and 4 °C for 2 h.

### 4.9. Cell Treatments

Macrophage-differentiated THP1 cells were incubated in duplicate with 10 µg/mL sEV-PBS or sEV-PAAF for 5 h, and the changes in the expression of inflammatory cytokines were evaluated through RT-PCR. In some experiments, cells were treated with 25 µM Chloroquine (Merck, Darmstadt, Germany) [19], 5 µM CU-CPT-22 (Calbiochem, San Diego, CA, USA) [20], 1 µg/mL CLI-095 (Invivogen, San Diego, CA, USA) [21], 1 µM MCC950 (Merck, Darmstadt, Germany) [22] or 1 µM FPS-ZM1 (Calbiochem, San Diego, CA, USA) [23]. For all experiments, *n* = 3.

### 4.10. RNA Isolation and qPCR

Total RNA from cells was extracted using the Trizol reagent (Invitrogen, Carlsbad, CA, USA) after 24 h of incubation with sEVs to ensure sufficient mRNA expression of the different cytokines evaluated. cDNA was synthesized using an iScript cDNA synthesis kit (Bio Rad, Hercules, CA, USA). A subsequent quantitative polymerase chain reaction (qPCR) was performed in a DNA Engine, Peltier Thermal Cycler (Bio-Rad, Hercules, CA, USA), using iTaqTM Universal SYBR^®^ Green Super mix and the corresponding primers (Supplementary Table S1). The specificity of the products was determined by melting curve analysis. The relative expression of target genes to GAPDH was calculated using the ΔC(t) formula.

### 4.11. Exosome Staining and Cell Uptake Analysis

sEVs were labeled with PKH26 Red Fluorescent Cell Linker Dye (Sigma, St. Louis, MO, USA). For cell uptake analysis, 10 μg/mL of PKH26 red-stained sEVs were added to the different cell cultures for 1 h, 3 h or 6 h. The cells were labeled with PKH67 Green Fluorescent Cell Linker Dye (Sigma, St. Louis, MO, USA). The cell nucleus was stained blue with Hoechst33342.

### 4.12. Fluorescence Microscopy

The cells were observed using an inverted Nikon Eclipse Ti2-E microscope (Nikon Instruments, Tokio, Japan) coupled with an Andor Dragonfly spinning disk unit and a high magnification objective, NIKON 60x NA 1.4, on a high-resolution camera (Zyla 4.2, 2.0 Andor, Oxford Instruments Company, Belfast, UK). Fusion software version 2.4.0.14 (Andor Oxford Instruments Company, Belfast, UK) was used for acquisition. Image processing and analysis were performed with Image J/Fifi 1.54 open-source software.

### 4.13. Digestion of Samples for Proteomic Analysis

The sEVs were incubated at 37 °C for 30 min with 2.5% trypsin in NH_4_HCO_3_ 20 mM to pre-digest the exposed proteins. Then, the samples were loaded on 100 kDa Amicon Ultra-0.5 filters (Sigma, St. Louis, MO, USA) and centrifuged for 10 min at 14,000× *g*. The flow though was recovered and incubated at 37 °C overnight with 2.5% trypsin. The reaction was quenched by adding 1% trifluoroacetic (TFA).

For PAAF characterization, 80 µg was lysed using 4.4% SDS, 0.1 M Tris/HCl (pH 7.5) and 0.1 M dithiothreitol (DTT), and digested using the filter-aided sample preparation (FASP) method as described in [24].

### 4.14. Proteomic Analysis

Samples were evaporated, redissolved in 5% methanol and 0.5% TFA, and analyzed using liquid chromatography coupled with mass spectrometry (LC-MS/MS) using a Fusion Lumos™ Tribrid mass spectrometer with a Dionex Ultimate 3000 chromatographic System (Thermo Scientific, Waltham, MA, USA). Each sample was loaded onto an Acclaim PepMap100 C18 Trap column (Thermo Scientific, Waltham, MA, USA), followed by separation on a NanoEase MZ HSS T3 column (Waters, Milford, MA, USA) with a 3–35% ACN gradient. The spectrometer operated in data-dependent mode, with full MS scans (range 350–1400) at a 120,000 resolution and MS/MS scans at a 30,000 resolution, using 28% collision energy for MS2 HCD.

Peptide identification was performed with Proteome Discoverer v3.0 using a 0.01% FDR. For sEV quantification, the database included *Homo sapiens*, *Rattus norvegicus* and common proteomics contaminants (MaxQuant), with trypsin as the enzyme (2 missed cleavages) and oxidation in methionine as a dynamic modification. For PAAF characterization, the database combined *Rattus norvegicus* and proteomics contaminants, with the carbamidomethylation of cysteines as a static modification. The precursor and fragment mass tolerance were set to 10 ppm and 0.02 Da. Gene ontology (GO) [25], KEGG Orthology [26] and BioGPS [27] were used for biological categorization of the proteins that were regulated in PAAF conditions.

### 4.15. Statistical Analysis

Statistical analysis was performed with GraphPad Prism 4.02 software. The data are presented as mean ± s.e.m. The data were analyzed using a two-tailed Student’s *t*-test for the comparison of two groups, and one-way analysis of variance (ANOVA) followed by Tukey’s post hoc test when comparing three groups. Statistical significance was considered when *p* < 0.05.

For the determination of rat-corona-specific proteins after PAAF incubation, we selected only proteins that could be unequivocally assigned to rats (proteins with unique peptides) and that were not present in vesicles treated with PBS alone. Abundance was expressed as a percentage of the total rat proteins appearing in the corona.

## 5. Conclusions

Our results show that the composition of the protein corona of sEVs undergoes significant and rapid changes depending on the microenvironment where they form. Using the inflammatory microenvironment generated in acute pancreatitis as a specific experimental model, we observed changes that underscore the general importance of the surrounding microenvironment in influencing both the cellular targets and the biological functions of sEVs. This highlights that the function of sEVs depends not only on their content, but also on the characteristics of the surrounding corona. Recognizing these dynamics could be essential for understanding the diverse roles of sEVs and for guiding the development of therapeutic strategies for diseases in which sEVs are implicated.

## Figures and Tables

**Table 1:** Hydrodynamic size and z-potential of sEVs treated with PBS or PAAF for 5 min.

sEV	Mean (nm)	Mode (nm)	z-Potential (mV)
PBS1	152.1 ± 2.5	123.1 ± 2.1	−33.27 ± 0.82
PAAF	158.6 ± 4.1	128.9 ± 3.3	−35.23 ± 1.39 *

* *p* < 0.05 PAAF vs. PBS.

**Table 2:** A list of rat proteins appearing on the surface of sEVs after incubation with PAAF. Only those that could be unequivocally assigned to a rat origin were included, and those that, due to the high homology between rats and humans, could not be guaranteed to come from the PAAF were not considered. Abundance is expressed as the % of total rat proteins incorporated into sEVs.

Uniprot Accession	Protein Name	Relative Abundance
P01026	Complement C3	10.1
P11517	Hemoglobin subunit beta-2	8.8
P24090	Alpha-2-HS-glycoprotein	6.8
P06399	Fibrinogen alpha chain	6.3
Q99PS8	Histidine-rich glycoprotein	5.9
Q9QX79	Fetuin-B	4.5
P04276	Vitamin D-binding protein	3.2
P62138	Serine/threonine-protein phosphatase PP1-alpha catalytic subunit	2.8
Q63416	Inter-alpha-trypsin inhibitor heavy chain H3	2.8
P13635	Ceruloplasmin	2.5
P00731	Carboxypeptidase	2.3
P54311	Guanine nucleotide-binding protein G(I)/G(S)/G(T) subunit beta-1	2.2
P36953	Afamin	2.2
P02767	Transthyretin	2.2
P04642	L-lactate dehydrogenase A chain	1.8
P04785	Protein disulfide-isomerase	1.7
P55018	Solute carrier family 12 member 3	1.6
P00774	Chymotrypsin-like elastase family member 2A	1.6
P62859	Small ribosomal subunit protein eS28	1.5
P10959	Carboxylesterase 1C	1.4
P20059	Hemopexin	1.3
P26644	Beta-2-glycoprotein 1	1.3
P07882	Bile salt-activated lipase	1.3
P00564	Creatine kinase M-type	1.2
P62836	Ras-related protein Rap-1A	1.2
Q66HD0	Endoplasmin	1.2
P01048	T-kininogen 1	1.1
P04916	Retinol-binding protein 4	1.0
P06866	Haptoglobin	1.0
P14141	Carbonic anhydrase 3	1.0
Q03626	Murinoglobulin-1	0.9
P31211	Corticosteroid-binding globulin	0.9
P14046	Alpha-1-inhibitor 3	0.9
P19939	Apolipoprotein C-I	0.9
P09006	Serine protease inhibitor A3N	0.8
P04638	Apolipoprotein A-II	0.8
Q05175	Brain acid soluble protein 1	0.8
P20761	Ig gamma-2B chain C region	0.7
P18418	Calreticulin	0.7
P35565	Calnexin	0.7
P05371	Clusterin	0.6
Q29RW1	Myosin-4	0.6
P18292	Prothrombin	0.5
P08649	Complement C4	0.5
D3ZF92	Tumor necrosis factor receptor superfamily member 21	0.5
Q6IMF3	Keratin, type II cytoskeletal 1	0.5
P02764	Alpha-1-acid glycoprotein	0.4
P20762	Ig gamma-2C chain C region	0.3
Q9JLJ3	4-trimethylaminobutyraldehyde dehydrogenase	0.3
Q9WUW3	Complement factor I	0.3
P15429	Beta-enolase	0.3
P48675	Desmin	0.3
P02401	Large ribosomal subunit protein P2	0.3
P27657	Pancreatic triacylglycerol lipase	0.2
P23764	Glutathione peroxidase 3	0.2
B2RZ37	Receptor expression-enhancing protein 5	0.2
Q5U2Q7	Eukaryotic peptide chain release factor subunit 1	0.2
O88994	Mitochondrial amidoxime reducing component 2	0.2
A6H909	TATA box-binding protein-like 2	0.2
Q71UF4	Histone-binding protein RBBP7	0.2
P55797	Apolipoprotein C-IV	0.2
G3V8D4	Apolipoprotein C-II	0.2
P04823	Interleukin-3	0.2
P55159	Serum paraoxonase/arylesterase 1	0.1
P24368	Peptidyl-prolyl cis-trans isomerase B	0.1
Q3MIE4	Synaptic vesicle membrane protein VAT-1 homolog	0.1
P63312	Thymosin beta-10	0.1
Q63556	Serine protease inhibitor A3M	0.1
P06302	Prothymosin alpha	0.1
Q6AYZ1	Tubulin alpha-1C chain	0.1
Q63862	Myosin-11	0.1
P56574	Isocitrate dehydrogenase [NADP], mitochondrial	0.1
P00684	Ribonuclease pancreatic beta-type	0.04

## Data Availability

The proteomic data are available in the PRoteomics IDEntifications (PRIDE) database with the accession number PXD057367.

## Supplementary Materials

Supporting information can be downloaded at: https://www.mdpi.com/article/10.3390/ijms252312969/s1.
