# Supplementary material for: The Microenvironment in an Experimental Model of Acute Pancreatitis Can Modify the Formation of the Protein Corona of sEVs, with Implications on Their Biological Function
Source: Int J Mol Sci. 2024 Dec 2;25(23):12969. doi: 10.3390/ijms252312969 (PMC11641761; doi:10.3390/ijms252312969)
Supplement: Supplementary file 1 [file ijms-25-12969-s001.zip › Supplementary Table S1.pdf]

Supplementary Table S1: Primers

Primer	Sequence forward primers (5'>3')	Sequence reverse primers (5'>3')
GAPDH	GATCATGAGCAATGCCTCCT	TGTGGTCATGAGTCGTTCCA
IL1β	GGACAAGCTGAGGAAGATGC	TCGTTATCCCATGTGTCGAA
TNF-α	GCCCATGTTGTAGCAAACC	GGCACCACCAACTGGTTATC
IL-6	TACCCCCAGGAGAAGATTCC	TTTTCTGCCAGTGCCTCTTT
MRC-1	GGATGGATGGCTCTGGTG	TCTGGTAGGAAACGCTGGTC
